# Supplementary material for: Pharmacokinetics and safety of oral glyburide in dogs with acute spinal cord injury
Source: PeerJ. 2018 Feb 26;6:e4387. doi: 10.7717/peerj.4387 (PMC5831157; doi:10.7717/peerj.4387)
Supplement: Table S1 [file peerj-06-4387-s001.docx]

**Supplementary Table 1:**

Summary of the mass spectrometer conditions and the compound parameters for glyburide and glipizide.

| **Compound** | **Polarity** | **Precursor m/z** | **Product m/z** | **Collision Energy (V)** |
| --- | --- | --- | --- | --- |
| Glipizide (ISTD) | Positive | 446.2 | 93.18 | 47 |
| Glipizide (ISTD) | Positive | 446.2 | 103.14 | 41 |
| Glipizide (ISTD) | Positive | 446.2 | 286.11 | 25 |
| Glyburide | Positive | 494.1 | 169.08 | 36 |
| Glyburide | Positive | 494.1 | 304.11 | 27 |
| Glyburide | Positive | 494.1 | 369.08 | 16 |
