# Supplementary material for: Pharmacokinetics and safety of oral glyburide in dogs with acute spinal cord injury
Source: PeerJ. 2018 Feb 26;6:e4387. doi: 10.7717/peerj.4387 (PMC5831157; doi:10.7717/peerj.4387)
Supplement: Table S2 [file peerj-06-4387-s002.docx]

| Time (hr) | Dog 1 | Dog 2 | Dog 3 | Dog 4 | Dog 5 |
| --- | --- | --- | --- | --- | --- |
| 0 | 112 | 75 | 104 | 121 | 177 |
| 1 | 120 | 84 | 111 | 115 | 118 |
| 2 | 120 | 50 | 118 | 108 | 166 |
| 3 | 125 | 53 | 99 | 123 | 144 |
| 4 | 119 | 114 | 97 | 157 | 166 |
| 6 | 92 | 113 | 138 | 88 | 144 |
| 8 | 109 | 79 | 97 | 102 | 126 |
| 10 | 89 | 93 | 93 | 106 | 110 |

**Supplementary Table 2:**

Blood glucose concentrations (mg/dL) in five dogs following oral dosing with 75 mcg/kg glyburide at time 0.
