# Supplementary material for: Pharmacokinetics and safety of oral glyburide in dogs with acute spinal cord injury
Source: PeerJ. 2018 Feb 26;6:e4387. doi: 10.7717/peerj.4387 (PMC5831157; doi:10.7717/peerj.4387)
Supplement: Table S3 — Note:a measurement of ‘0’ is given for any value below the lower level of detection of 0.5 ng/mL. [file peerj-06-4387-s003.docx]

| **Time (hrs)** | **Dog 1** | **Dog 2** | **Dog 3** | **Dog 4** | **Dog 5** | **Dog 6** |
| --- | --- | --- | --- | --- | --- | --- |
|  |  |  |  |  |  |  |
| **0** | 0 | 0 | 0 | 0 | 0 | 0 |
| **1** | 2.3 | 1.8 | 2.7 | 18.2 | 1.3 | 0 |
| **2** | 3.1 | 3.8 | 15.6 | 32.2 | 2.1 | 1.5 |
| **3** | 5.8 | 5.4 | 20.8 | 21.3 | 2.7 | 2 |
| **4** | 6.9 | 6.2 | 34.1 | 12.5 | 3 | 1.9 |
| **5** | 5.0 | 6.5 | 22.3 | 22.1 | 7.9 | 1.7 |
| **6** | 12.1 | 6.3 | 20.7 | 18.4 | 12.1 | 1.6 |
| **8** | 13.0 | 16.1 | 32.8 | 6.9 | 13.5 | 2.5 |
| **10** | 9.0 | 26.2 | 25.4 | 9 | 36.7 | 3.4 |
| **12** | 12.6 | 24.3 | 54.3 | 6.7 | 44.2 | 3.9 |
| **14** | - | 23.3 | 35.2 | 4.9 | 19.1 | 7.2 |
| **16** | 20.9 | 16.9 | 15.2 | 3.2 | 20.9 | 6.4 |
| **24** | 6 | 11.6 | 7.1 | 1.9 | 6 | 9.7 |

**Supplementary Table 3: Plasma glyburide concentrations in each of six dogs that received 75mcg/kg oral glyburide at time 0.**

**Note:** a measurement of ‘0’ is given for any value below the lower level of detection of 0.5ng/mL
