# Supplementary material for: Pharmacokinetics and safety of oral glyburide in dogs with acute spinal cord injury
Source: PeerJ. 2018 Feb 26;6:e4387. doi: 10.7717/peerj.4387 (PMC5831157; doi:10.7717/peerj.4387)
Supplement: Table S4 — Notes:* indicates test samples obtained after intravenous glucose supplementation and so not used in this analysis; - indicates sample not analyzed or not recorded at this time point. [file peerj-06-4387-s004.docx]

| Time (hrs) | Dog 1 | | Dog 2 | | Dog 3 | | Dog 4 | | Dog 5 | | Dog 6 | |
| --- | --- | --- | --- | --- | --- | --- | --- | --- | --- | --- | --- | --- |
|  | Gly | Glu | Gly | Glu | Gly | Glu | Gly | Glu | Gly | Glu | Gly | Glu |
| 0 | 0 | 81 | 0 | 114 | 0 | 74 | 0 | 84 | 0 | 96 | 0 | 123 |
| 1 | 1.8 | 88 | 2.7 | 149 | 18.2 | 82 | 2.3 | 95 | 1.3 | 95 | 0 | 111 |
| 2 | 3.8 | 74 | 15.6 | 107 | 32.2 | 102 | 3.1 | 80 | 2.1 | 81 | 1.5 | 109 |
| 3 | 5.4 | 62 | 20.8 | 74 | 21.3 | 105 | 5.8 | 74 | 2.7 | 74 | 2 | 121 |
| 4 | 6.2 | 73 | 34.1 | 127 | 12.5 | 92 | 6.9 | 50 | 3 | 65 | 1.9 | 109 |
| 5 | 6.5 | 103 | 22.3 | 119 | 22.1 | 76 | 5.0 | * | 7.9 | 88 | 1.7 | 128 |
| 6 | 6.3 | 107 | 20.7 | 111 | 18.4 | 67 | 12.1 | * | 12.1 | 90 | 1.6 | 99 |
| 8 | 16.1 | 69 | 32.8 | 106 | 6.9 | 81 | 13.0 | * | 13.5 | 67 | 2.5 | 324 |
| 10 | 26.2 | 63 | 25.4 | 92 | 9.0 | 63 | 9.0 | * | 36.7 | 72 | 3.4 | 196 |
| 12 | 24.3 | 68 | 54.3 | 146 | 6.7 | 83 | 12.6 | * | 44.2 | 77 | 3.9 | 106 |
| 14 | 23.3 | 67 | 35.2 | 138 | 4.9 | 101 | - | * | 19.1 | 69 | 7.2 | 94 |
| 16 | 16.9 | 60 | 15.2 | - | 3.2 | 111 | 20.9 | * | 20.9 | 81 | 6.4 | 124 |
| 24 | 11.6 | 72 | 7.1 | 139 | 1.9 | 125 | 6.0 | * | 6 | 82 | 9.7 | 101 |

**Supplementary Table 4: Whole blood glucose and plasma glyburide in six dogs that received 75mcg/kg glyburide orally at time 0.**

**Notes:** * indicates test samples obtained after intravenous glucose supplementation and so not used in this analysis; - indicates sample not analyzed or not recorded at this time point.
